# Niche matters: The comparison between bone marrow stem cells and endometrial stem cells and stromal fibroblasts reveal distinct migration and cytokine profiles in response to inflammatory stimulus

**DOI:** 10.1371/journal.pone.0175986

**Published:** 2017-04-18

**Authors:** Masuma Khatun, Anna Sorjamaa, Marika Kangasniemi, Meeri Sutinen, Tuula Salo, Annikki Liakka, Petri Lehenkari, Juha S. Tapanainen, Olli Vuolteenaho, Joseph C. Chen, Siri Lehtonen, Terhi T. Piltonen

**Affiliations:** 1 Department of Obstetrics and Gynecology, PEDEGO Research Unit, Medical Research Center, University of Oulu and Oulu University Hospital, Oulu, Finland; 2 Cancer and Translational Medicine Research Unit, Medical Research Center, University of Oulu and Oulu University Hospital, Oulu, Finland; 3 Department of Pathology, Medical Research Center, University of Oulu and Oulu University Hospital, Oulu, Finland; 4 Department of Anatomy and Department of Internal Medicine, Medical Research Center, University of Oulu and Oulu University Hospital, Oulu, Finland; 5 Department of Obstetrics and Gynecology, University of Helsinki and Helsinki University Central Hospital, Helsinki, Finland; 6 Research Unit of Biomedicine, University of Oulu, Oulu, Finland; 7 Department of Obstetrics, Gynecology and Reproductive Sciences, University of California, San Francisco, United States of America; Universita degli Studi di Torino, ITALY

## Abstract

**Objective:**

Intrinsic inflammatory characteristics play a pivotal role in stem cell recruitment and homing through migration where the subsequent change in niche has been shown to alter these characteristics. The bone marrow mesenchymal stem cells (bmMSCs) have been demonstrated to migrate to the endometrium contributing to the stem cell reservoir and regeneration of endometrial tissue. Thus, the aim of the present study was to compare the inflammation-driven migration and cytokine secretion profile of human bmMSCs to endometrial mesenchymal stem cells (eMSCs) and endometrial fibroblasts (eSFs).

**Materials and methods:**

The bmMSCs were isolated from bone marrow aspirates through culturing, whereas eMSCs and eSFs were FACS-isolated. All cell types were tested for their surface marker, proliferation profiles and migration properties towards serum and inflammatory attractants. The cytokine/chemokine secretion profile of 35 targets was analysed in each cell type at basal level along with lipopolysaccharide (LPS)-induced state.

**Results:**

Both stem cell types, bmMSCs and eMSCs, presented with similar stem cell surface marker profiles as well as possessed high proliferation and migration potential compared to eSFs. In multiplex assays, the secretion of 16 cytokine targets was detected and LPS stimulation expanded the cytokine secretion pattern by triggering the secretion of several targets. The bmMSCs exhibited higher cytokine secretion of vascular endothelial growth factor (VEGF)-A, stromal cell-derived factor-1 alpha (SDF)-1α, interleukin-1 receptor antagonist (IL-1RA), IL-6, interferon-gamma inducible protein (IP)-10, monocyte chemoattractant protein (MCP)-1, macrophage inflammatory protein (MIP)1α and RANTES compared to eMSCs and/or eSFs after stimulation with LPS. The basal IL-8 secretion was higher in both endometrial cell types compared to bmMSCs.

**Conclusion:**

Our results highlight that similar to bmMSCs, the eMSCs possess high migration activity while the differentiation process towards stromal fibroblasts seemed to result in loss of stem cell surface markers, minimal migration activity and a subtler cytokine profile likely contributing to normal endometrial function.

## Introduction

The human endometrium has a unique ability to regenerate rapidly, increasing its thickness from 2–4 mm in the early proliferative phase to 10–15 mm by the end of the secretory phase [1,2]. The growth of the endometrial tissue is under steroid hormone (estradiol [E2] and progesterone [P4]) control, where the monthly cycles of growth, differentiation and shedding occur in response to ovarian hormonal fluctuations [3]. With blastocyst implantation, the endometrium is challenged with immune tolerance, the regulation of trophoblast invasion and vasculature formation, in which a balanced hormonal and immune environment, the niche is crucial for successful and healthy pregnancy [4–6]. In a non-conception cycle, the endometrium goes through a complex inflammatory process involving cell drift and immune cell migration leading to the activation of degradative enzymes and apoptosis, subsequent tissue breakdowns and menstruation. Simultaneously, the molecular processes ensuring tissue regeneration, revascularization and histoarchitectural development are initiated, most likely through inflammatory triggers related to menstruation-induced hypoxia, to prepare the endometrium for the next menstrual cycle [7].

Endometrial mesenchymal stem cells (eMSCs) have been reported to reside in the perivascular space in the human endometrium, most likely contributing to the monthly regeneration and repair of this tissue [1,3,8]. These very rare adult stem cells are defined by their functional properties, such as substantial self-renewal, high proliferative potential and the ability to differentiate into one or more cell lineages, including osteocytes, adipocytes and chondrocytes [1,8]. The global gene profile analysis has revealed that eMSCs and endometrial stromal fibroblasts (eSFs) have similar genomic signatures, suggesting that eMSCs are progenitors of eSFs, the most common cell type in the endometrium [2,9]. The mesenchymal stem cells of different tissues have been described as having migration activity towards the site of injury in response to secreted cytokines and chemokines [10,11]. In terms of endometrium repair, several studies have suggested that eMSCs have a bone marrow origin: signals related to tissue damage (menstruation) initiate bone marrow mesenchymal stem cells’ (bmMSCs) migration to the endometrium, where they differentiate into eMSCs, contributing to the endometrial stem cell reservoir and thereby endometrial regeneration [12–14].

In the human endometrium, cytokine/chemokine secretion is regulated by hormonal fluctuations, which is one of the key factors orchestrating implantation and monthly endometrial regeneration [15,16]. Steroid hormone withdrawal during the late secretory phase leads to hypoxia, the initiation of several inflammatory processes including leucocyte recruitment and increased synthesis of cytokines like interleukin 1β (IL-1β), and other inflammatory modulators within the cells most likely providing the key event for homing the bmMSCs in the endometrium [15,17,18]. Interestingly, previous studies have suggested that mechanisms for the initiation and regulation of bmMSCs’ migration to different tissues involve the secretion of distinct sets or even individual cytokines [19–24]. In the human endometrium, IL-1β, a major pro-inflammatory cytokine regulating many of the endometrial functions, may be considered as a potential trigger for bmMSCs’ recruitment due to its expression response to hypoxia [25–28]. On the other hand, the endometrial stem cells have also been shown to possess migratory abilities [29,30]. However, to date, comparative studies assessing the migratory characteristics of bmMSCs in response to inflammatory triggers compared to the assumable endometrial progeny, eMSCs and further down in the line the eSFs, are scarce.

Traditionally, MSCs are thought to regulate the immune response and exhibit niche-dependent pro- and anti-inflammatory properties via cytokine and chemokine secretion [31]. As the bmMSCs serve as a source for stem cells for several tissues, it is acquired that upon cell migration and change in niche, also the cytokine secretion profile and thereby paracrine signalling is changed. Thus, the new cytokine profile may also have an effect on migration signalling of this tissue [22]. Indeed, previous studies have assessed the cytokine secretion patterns between MSCs of different tissues [31], however, the endometrium as a relatively new source for MSCs remains poorly characterized, especially regarding the comparative data between bmMSCs and different endometrial cell populations. Furthermore, it is well recognized that the basal cytokine patterns do not correlate with the total secretion potential of the cells [32–34]. In fact, the majority of the previous studies describe only the basal cytokine secretion profiles but not the profiles under stimulation.

Given the plausible role of bmMSCs in endometrial regeneration through migration and the differences in bone marrow and endometrial niche the first aim was to investigate the surface marker signature and IL-1β triggered migration of bmMSCs and compare these characteristics to their assumed endometrial progeny, the eMSCs and eSFs. Secondly, we also compared for the first time the basal and stimulated cytokine secretion profiles of bmMSCs, eMSCs and eSFs in the same study setting in order to reveal their paracrine properties that may relate to endometrial regeneration and migration signalling but also to normal endometrial function. Our results show that indeed there is a specific surface marker as well as inflammation driven migration profile for the bmMSCs, eMSCs and eSFs enabling more detailed characterization of these three cell types. Moreover, the results suggest a niche effect on the cytokine secretion characteristics that was shown as distinct cytokine secretion pattern in bmMSCs compared with endometrial eMSCs and eSFs.

## Materials and methods

### Study subjects

Bone marrow aspirates (n = 12) were obtained from fertile-aged women undergoing surgery for scoliosis in the Department of Paediatric Surgery, Oulu University Hospital, Finland. The endometrial tissue biopsy samples (n = 15, whole uterus n = 4) were obtained from fertile-aged women undergoing surgery for benign gynaecological conditions, or from healthy volunteers in the Department of Obstetrics and Gynaecology, Oulu University Hospital, Finland. One full thickness endometrial sample from endometriosis was used in co-localization in immunofluorescence and was not used in any other experiments. Clinical data on the study participants is shown in Table 1. Informed written consent was obtained from all participants in accordance with the guidelines of the Declaration of Helsinki, and the Ethics Committee of Oulu University Hospital approved the study.

**Table 1:** Clinical characteristics of the study subjects.

ID	Obtained cell	Age (years)	BMI	Histology	Diagnosis	Sample Collection	Hormonal Medication	Smoking
**Endo 1** ^a^^,^ ^b^	eMSC, eSF	42	19.6	PE	Myoma	Pipelle	No	No
**Endo 2** ^a^^,^ ^b^	eMSC, eSF	39	22.3	SE	Polysuscipion	Pipelle	No	No
**Endo 3** ^a^^,^ ^b^	eMSC, eSF	40	26.3	PE	Myoma, Menorrhagia	Pipelle	No	No
**Endo 4** ^a^^,^ ^b^	eMSC, eSF	23	23.1	SE	Hypertophic Labia	Pipelle	No	Yes
**Endo 5** ^b^	eMSC, eSF	44	33.5	SE	Myoma	Pipelle	No	No
**Endo 6** ^b^^,^ ^c^^,^ ^e^	eMSC, eSF	41	19.3	PE	Left ovarian Cyst	Pipelle	No	No
**Endo 7** ^b^^,^ ^c^^,^ ^e^	eMSC, eSF	44	24.4	SE	Endometrial Polyp	Pipelle	No	No
**Endo 8** ^b^^,^ ^c^^,^ ^e^	eMSC, eSF	22	18.8	SE	Left ovarian Cyst	Pipelle	No	Yes
**Endo 9** ^c^	eMSC, eSF	40	26.1	SE	Left ovarian Cyst	Pipelle	No	No
**Endo 10** ^c^	eMSC, eSF	37	24.6	PE	Myoma	Pipelle	No	Yes
**Endo 11** ^b^	eMSC, eSF	44	36.1	N/A	Pelvic floor prolapse	Pipelle	No	No
**Endo 12** ^b^^,^ ^e^	eMSC, eSF	41	25.6	PE	Volunteer	Pipelle	No	No
**Endo 13** ^b^	eMSC, eSF	34	24.9	PE	Volunteer	Pipelle	No	No
**Endo 14** ^b^	eMSC, eSF	42	38.4	PE	Volunteer	Pipelle	No	No
**Endo 15** ^d^	N/A	42	27.2	SE	Menorrhagia	Uterus	No	Yes
**Endo 16** ^d^	N/A	40	24.2	DQ	Myoma, Menorrhagia	Uterus	No	Yes
**Endo 17** ^d^	N/A	43	30.1	PE	Menorrhagia	Uterus	No	No
**Endo 18** ^d^	N/A	39	24	PE	Endometriosis	Uterus	No	No
**Endo 19** ^e^	eMSC	36	20.5	SE	Cysta dermoidea ovarii	Pipelle	No	No
**BM 1** ^a^^,^ ^b^^,^ ^c^	bmMSC	36	N/A	N/A	Scoliosis	BMA	N/A	N/A
**BM 2** ^a^^,^ ^b^^,^ ^c^	bmMSC	34	N/A	N/A	Scoliosis	BMA	N/A	N/A
**BM 3** ^a^^,^ ^b^^,^ ^c^	bmMSC	26	N/A	N/A	Scoliosis	BMA	N/A	N/A
**BM 4** ^a^^,^ ^b^^,^ ^c^	bmMSC	37	N/A	N/A	Scoliosis	BMA	N/A	N/A
**BM 5** ^a^^,^ ^b^^,^ ^c^	bmMSC	44	N/A	N/A	Scoliosis	BMA	N/A	N/A
**BM 6** ^a^^,^ ^b^^,^ ^c^	bmMSC	46	N/A	N/A	Scoliosis	BMA	N/A	N/A
**BM 7** ^a^^,^ ^b^^,^ ^c^	bmMSC	36	N/A	N/A	Scoliosis	BMA	N/A	N/A
**BM 8** ^a^^,^ ^b^^,^ ^c^	bmMSC	45	N/A	N/A	Scoliosis	BMA	N/A	N/A
**BM 9** ^e^	bmMSC	16	N/A	N/A	Scoliosis	BMA	N/A	N/A
**BM 10** ^e^	bmMSC	16	N/A	N/A	Scoliosis	BMA	N/A	N/A
**BM 11** ^e^	bmMSC	16	N/A	N/A	Scoliosis	BMA	N/A	N/A
**BM 12** ^e^	bmMSC	16	N/A	N/A	Scoliosis	BMA	N/A	N/A

eMSC, endometrial mesenchymal stem cell; eSF, endometrial stromal fibroblast; bmMSC, bone marrow mesenchymal stem cell

PE, proliferative phase; SE, secretory phase; DQ, desquamation; BMA, bone marrow aspirate; N/A, Not Acquired

^a^ Sample used in surface marker analysis

^b^ Sample used in differentiation studies

^c^ Sample used in migration and assays

^d^ Sample used in immunofluorescence

^e^ Sample used in Luminex Multiplex assay

### Tissue processing and the Fluorescence-Activated Cell Sorting (FACS) of endometrial cell populations

Endometrial samples were divided into two groups and processed separately for FACS and for histological examination, as described earlier [35]. Briefly, for FACS the tissues were digested with collagenase type I (Sigma) at 6.4 mg/ml and hyaluronidase (Sigma) at 125 U/ml and filtered with a 40 μm cell strainer to separate single cells. Contaminating red cells were lysed with 0.155 M of NH_4_Cl, 0.1 M of KHCO_3_ and 0.1 mM EDTA, at a pH of 7.3 (Sigma), and the dissociated cellular elements were treated with DNase (Mediq) at 4 mg/ml.

For FACS isolation, single-cell fragments of endometrial samples (n = 15) were blocked and labelled with the following fluorochrome-conjugated antibodies (BD Biosciences) in phosphate-buffered saline (PBS) containing 10% human serum and 1% BSA for an hour: cluster of differentiation (CD) 45 (phycoerythrin-Cy7 anti-CD45) at a 1:20 dilution to remove contaminating leucocytes; epithelial cell adhesion molecule (EPCAM; allophycocyanin anti EPCAM) at a 1:20 dilution) to label contaminating endometrial epithelial cells, cluster of differentiation 146 (CD146, melanoma cell adhesion molecule [MCAM], fluorescein isothiocyanate anti-MCAM) at a 1:5 dilution to label perivascular cells; β-type platelet-derived growth factor receptor (PDGFRβ; phycoerythrin anti-PDGFRβ) at a 1:5 dilution to label eSFs (PDGFRβ+/CD146-). Endometrial MSCs were sorted using double labelling with PDGFRβ and CD146 antibodies, both at 1:5 dilutions. DAPI staining was used for distinguishing live/dead cells (NucBlue^®^, Life Technologies). The FACS-isolation protocol has previously been shown to produce pure cell populations [2,36].

### Cell culturing and the isolation of bmMSC populations

FACS-isolated eMSCs and eSFs were plated with a density of 1000–2000 cells in 10-cm plate for eMSCs and with 200,000 cells in 10-cm plate for eSFs. Similarly to previous study, no epithelial cell contamination were found in the eMSC or eSF cultures after careful monitoring during the entire culture period [9,37]. eMSCs were cultured in phenol red-free high-glucose DMEM growth medium (Life Technologies) supplemented with 25% MCDB-105 (Sigma), 0.676 mM of sodium pyruvate (Sigma), 10% charcoal-stripped foetal bovine serum (FBS; Seralab, UK), 1% penicillin-streptomycin mix (Life Technologies) and 50 μg/ml of gentamycin (Life Technologies). In addition, 25 μg/ml of basic fibroblast growth factor (FGFb; Life Technologies) was added to eMSC primary cultures. Growth media (without FGFb) was used in the culturing of the eSF with 5 μg/ml of insulin (Sigma). Growth media was changed every 2–3 days and the confluency of eMSCs was maintained below 80% during culturing. The eMSCs and eSFs were cultured up to passage two.

For the bmMSC culture, a bone marrow aspirate was plated in alpha modified essential growth medium (Sigma) supplemented with 20 mM of Hepes, 2 mM of L-glutamine (Sigma), 10% heat-inactivated FBS (Bioclear), 10,000 U penicillin/ml and 10 mg/ml of streptomycin (Sigma). After two days, unattached cells were removed. Half of the growth media was changed twice per week. The confluency of bmMSCs was maintained below 80% [38]. For the final analysis and experiments, all the cultured cells were washed with PBS and adherent cells were detached using trypsin-EDTA solution (Gibco).

### Co-localization of eMSC with markers for PDGFRβ+/ CD146+

Endometrial tissue sections obtained from hysterectomies (n = 4) were embedded in a Tissue-Tek Optimal Cutting Temperature (OCT) compound (Miles Laboratories), frozen in liquid nitrogen and stored at -80°C. Perpendicular tissue samples containing both endometrium and myometrium were taken from the posterior uterine wall. Histological evaluation was performed by a pathologist.

Frozen sections (~6μm) were fixed in methanol (-20°C) for 10 min and permeabilized for 10 min using 0.5% Tween20 in 0.01 M of PBS (Sigma) rinse solution. The sections were blocked in antibody dilution buffer (two parts 0.01 M of PBS, 2% BSA and 0.5% Tween20, and one part glycerol; (Sigma)) containing 10% normal goat serum (Vector laboratories) for 1h at room temperature (RT). Sections were washed with PBS/Tween and incubated with anti-CD146 (Abcam, 5 μg/ml) for 1 h at RT and then overnight at +4°C. After washing, the sections were incubated with Alexa fluor 488 goat anti-mouse antibodies (Molecular Probes, 8 μg/ml) for 4 h and washed in rinsing solution. The sections were then incubated for 1 h at RT and then overnight at +4°C with anti-PDGFRβ (Abcam, 5μg/ml). After washing, the sections were incubated with Alexa fluor 594 goat anti-rabbit antibody (Molecular Probes, 8 μg/ml) for 4 h at +4°C and washed in rinsing solution at RT. The sections were then mounted in a Vectashield antifade mounting medium containing DAPI (Vector, H-1200). Images were captured using an AxioCam MRm microscope fitted with high-resolution Imager.D2 software (Carl Zeiss).

### Stem cell surface marker analysis using flow cytometry

Selected cell surface markers were identified by flow cytometry (FACS Calibur). Cultured eMSCs, eSFs and bmMSCs (n = 4/cell type) were incubated for 20 min at RT with the following antibodies at a dilution of 1:50: CD105, CD73, CD90, human leucocyte antigen (HLA) ABC, CD44, CD49d, CD49f, HLA-DR1, CD34, CD45, CD14, CD19, c-MET, PDGFRβ/CD140b or CD146 (CD90 from The Cell Technologies, all others from BD Biosciences). Unbound antibodies were washed off and the cells were analysed using flow cytometry. Stain intensities were recorded as follows: -, negative; +, <35%. ++, 35–70%; +++, 95–100%, indicating the proportion of cells staining positively.

### Proliferation/migration assessment of cultured cells using scratch assays

Proliferation/migration potential was analysed using a cell culture scratch assay protocol. bmMSCs, eMSCs and eSFs (n = 4) were seeded into 24-well plates (100,000 cells/ well). Immediately after reaching confluency on day 3, crisscross scratches were made with p10 pipette tips and the medium was replaced with a culture medium containing 2% FBS. The scratched area was monitored and photographed regularly using an EVOS Digital Inverted Microscope (Advanced Microscopy Group, Bothell, WA, USA), starting immediately after the scratching and ending when one of the cell types showed 100% repair of the scratched area. The empty areas were measured using ImageJ software (version 1.50), and the results were calculated as a percentage of the area closed by the cell growth.

### Transwell^®^ migration assays

To study vertical cell migration, Corning Transwell^®^ 8.0 μm polycarbonate membrane 6.5 mm inserts (24-well plate, Costar 3422, Sigma) were used. bmMSCs, eMSCs or eSFs (n = 4/cell type) were seeded (20,000 cells/well) into the upper chamber in 2% FBS culture medium in duplicate in several repetitions. In addition, 10% FBS culture medium or 2% FBS medium with IL-1β (Sigma) at 10 ng/ml were added to the lower chamber as attractants; 2% FBS culture medium in both the upper and lower chamber was used as the baseline. After 24, 48, 72 and 96 h of migration, the inserts were fixed with 4% paraformaldehyde and stained with 1% toluidine blue + 1% sodium tetraborate. Non-migrating cells from the top of the membrane were removed using a cotton swab. The dye attached to the migrated cells was eluted by dipping the inserts into 1% sodium dodecyl sulphate (Roche), followed by measuring the absorbance at 650 nm using a Wallac Victor 2^™^ Multilabel counter.

### Lipopolysaccharide (LPS) stimulation and Luminex multiplex assays

For the cytokine secretion analysis, eMSCs, eSFs and bmMSCs (n = 4/cell type) were plated to 24-well plates with a seeding density of 20,000–50,000 cells/well. After reaching 80% confluency, the cells were switched to growth media containing 2% FBS. After 48 h, the cells were challenged with 10 ng/ml of LPS supplemented in 2% FBS growth media, keeping control cells in 2% FBS growth media. After 24 h of LPS treatment, the cell culture media was collected and the cells were harvested from the wells with trypsin-EDTA solution (Gibco).

Cytokines, chemokines and related proteins like Eotaxin, granulocyte macrophage colony stimulating factor (GM-CSF), melanoma growth stimulatory activity alpha (GRO-α), interferon alpha 1 (FNA1), interferon gamma (FNG), interleukin (IL)-10, IL-12p70, IL-13, IL-15, IL-17A, IL-18, IL-1a, IL-1β, IL-1RA, IL-2, IL-21, IL-22, IL-23, IL-27, IL-31, IL-4, IL-5, IL-6, IL-7, IL-8, IL-9, interferon-gamma inducible protein-10 (IP-10), monocyte chemoattractant protein (MCP-1), macrophage inflammatory protein 1-alpha (MIP-1α), macrophage inflammatory protein 1-beta (MIP-1β), regulated on activation, normal T-cell expressed and secreted (RANTES), stromal cell-derived factor 1 alpha (SDF-1α), tumour necrosis factor-alpha (TNF-α), tumour necrosis factor-beta (TNF-β) and vascular endothelial growth factor VEGF-A) were quantified with eBioscience ProcartaPlex Human Cytokine & Chemokine 34plex supplemented with VEGF-A Simplex kit using a Luminex MagPix system and Luminex xPonent Software. The culture medium samples were assayed both undiluted and after a 1:10 dilution. Milliplex Analyst software (VigeneTech) was used for the multiplex assay data extraction. The coefficient of determination (R2) of the 5-parameter logistic regression standard curves were between 0.999 and 1.000 in all assays, and the standard curve CVs were between 0.15% (MIP-1β) and 2.7% (IL-27). Total protein was isolated from the cells using Tri Reagent for RNA, DNA and protein isolation (Sigma), and the results were normalized (using a dilution factor) against total protein measured with a Direct Detect^®^ Infrared Spectrometer (Merck Millipore).

## Statistical analysis

The results are presented as the mean ± SD. Analysis of variance between groups was performed with one-way ANOVA, using a nonparametric approach when appropriate. T-tests and nonparametric Mann-Whitney, when appropriate, were carried out as post-hoc tests. P-values < 0.05 were considered statistically significant. IBM SPSS statistics software version 22.0 and Graphpad prism 6 was used for all statistical analyses.

## Ethical approval

The sample collection was approved by the ethics committee of Oulu University Hospital (PPSHP) under the statement number 22/2013 at 18/02/2013. The data did not include any identification information from the patients and was handled only by the members of the research team.

## Results

### FACS isolation and co-localization of (PDGFRβ+/CD146+) markers

**Fig 1:**
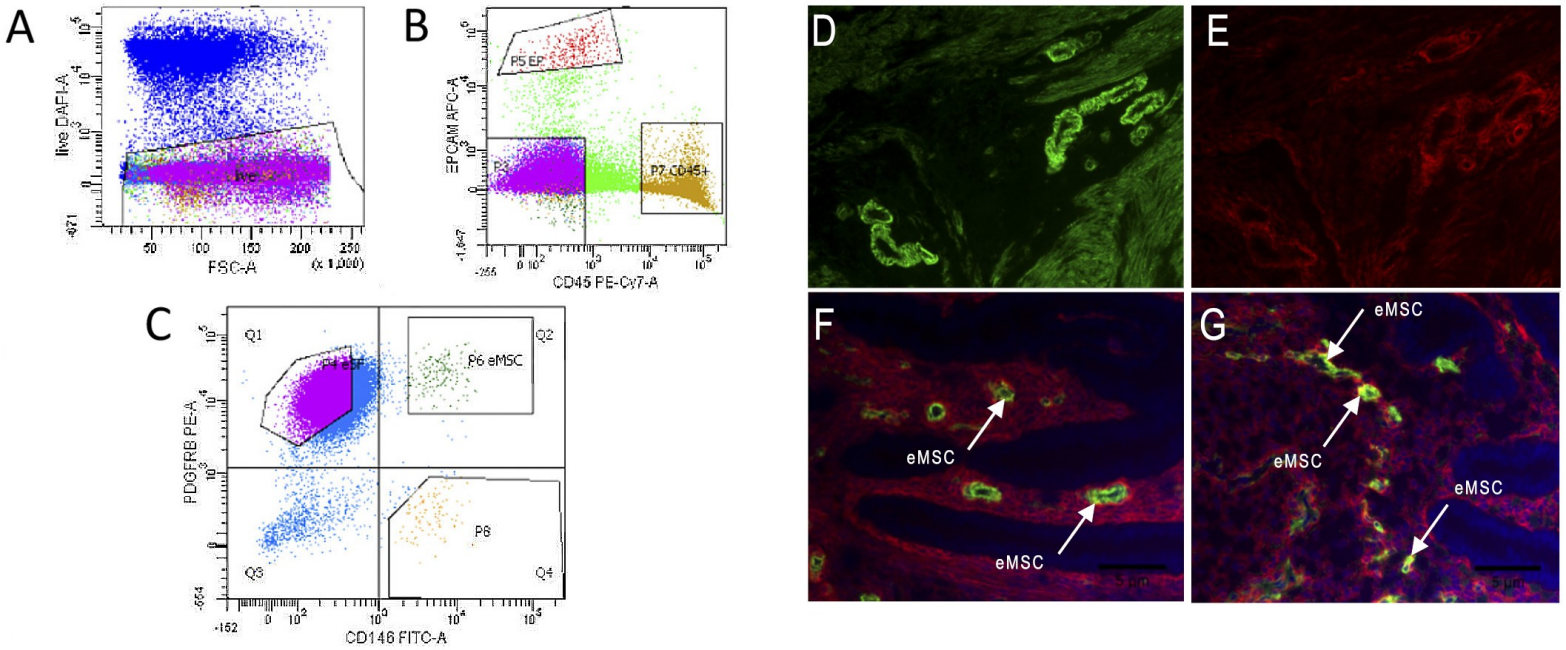
FACS sorting of eMSCs, eSFs and localization of eMSCs in the human endometrium. (A) Endometrial cell populations were isolated from a single live cell fraction using DAPI stain. (B) Contaminating epithelial cells (EP) (Epcam^+^) and leucocytes (CD45^+^) were gated out. (C) eSFs positive for β-type platelet-derived growth factor receptor (PDGFRβ) and negative for cluster of differentiation 146 (CD146, melanoma cell adhesion molecule [MCAM]) and eMSCs (PDGFRβ^+^/CD146^+^) were isolated (areas Q1 and Q2). (D-G) eMSCs were identified in the perivascular space in the basal layer of proliferative phase human endometrium by identifying co-localization of CD146 (green) and PDGFRβ (red) expression (white arrows). Scale bar 5 μm.

In FACS analysis, epithelial cells (epithelial cell adhesion molecule (EPCAM+)), leucocytes (CD45+) and endothelial cells (β-type platelet-derived growth factor receptor (PDGFRβ-) /CD146+)) were gated out (Fig 1A and 1B), and eMSCs and eSFs were isolated according to their staining patterns (eSFs, PDGFRβ+/CD146-; eMSCs, PDGFRβ+/CD146+; Fig 1C). The eMSCs clustered separately from the eSFs, indicating the purity of downstream cell culture populations. In immunofluorescence, eMSCs were located in the perivascular space in the basal layer (Fig 1D–1G), as well as in the functional layer (data not shown).

### BmMSCs and eMSCs present with similar stem cell surface marker profiles

**Fig 2:**
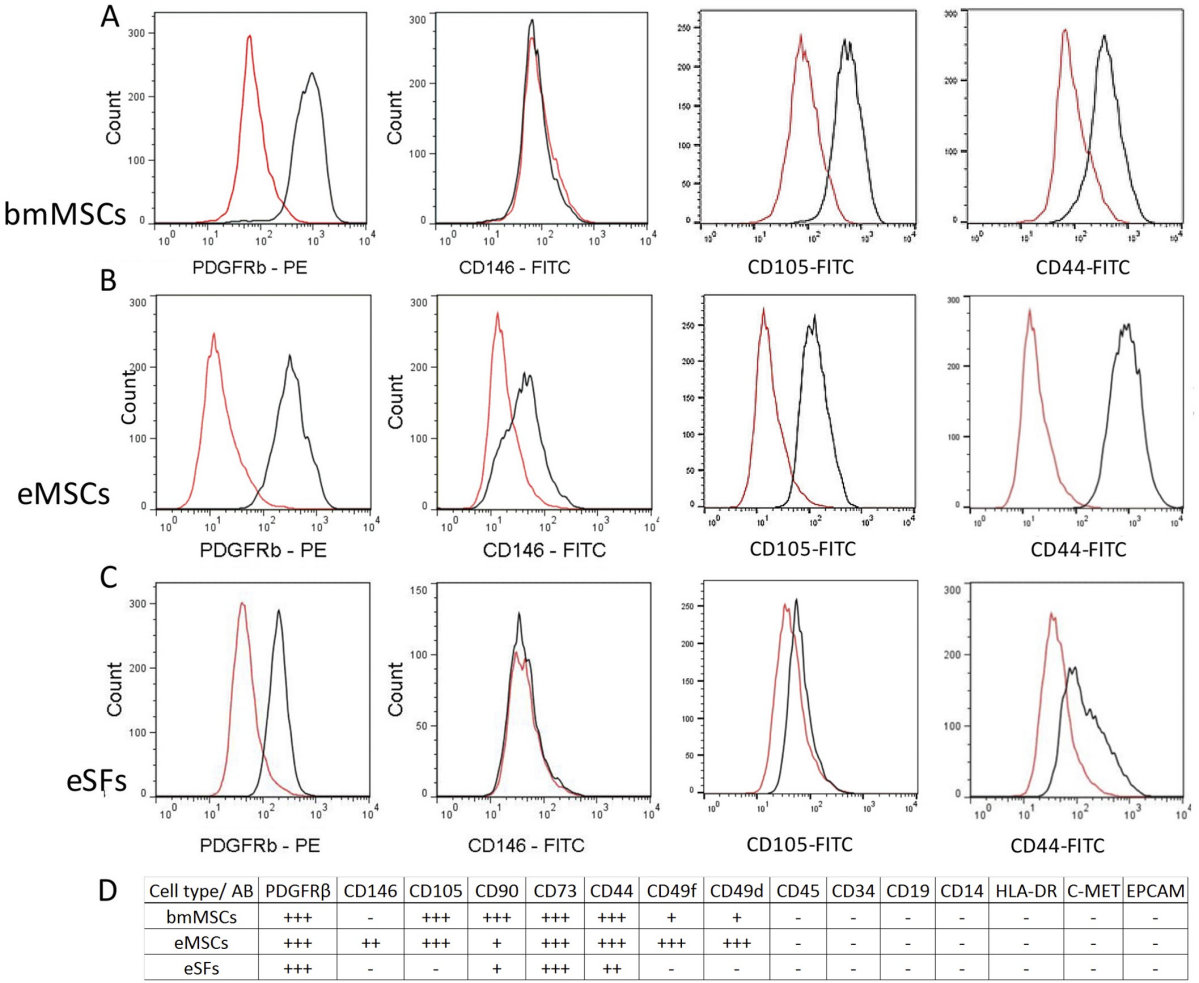
Surface marker analysis of bmMSCs, eMSCs and eSFs. (A, C) bmMSCs and eSFs were positive only for PDGFRβ and negative for CD146. bmMSCs were also strongly positive for CD105 and CD44, whereas eSFs were negative for CD105 and showed only low expression of CD44. (B) eMSCs presented with double staining for PDGFRβ and CD146 and were strongly positive for CD105 and CD44. (D) A summary table of surface marker analysis.

Both bmMSCs and eMSCs presented with a typical stem cell surface antigen profile showing positive staining for CD105, CD90, CD73, CD44, CD49d and CD49f. All cell types were negative for haematopoietic cell markers CD45, CD34, CD19, CD14, human lymphocyte antigen-DR (HLA-DR), the carcinoma cell marker (c-MET) and EPCAM (Fig 2D). The surface marker analysis of passage 2 cultured cells revealed that eMSCs were still strongly positive for PDGFRβ and CD146, whereas bmMSCs and eSFs were positive only for PDGFRβ (Fig 2A–2C).

### BmMSCs and eMSCs demonstrate high proliferation potential and a robust migration response to inflammatory attractant

**Fig 3:**
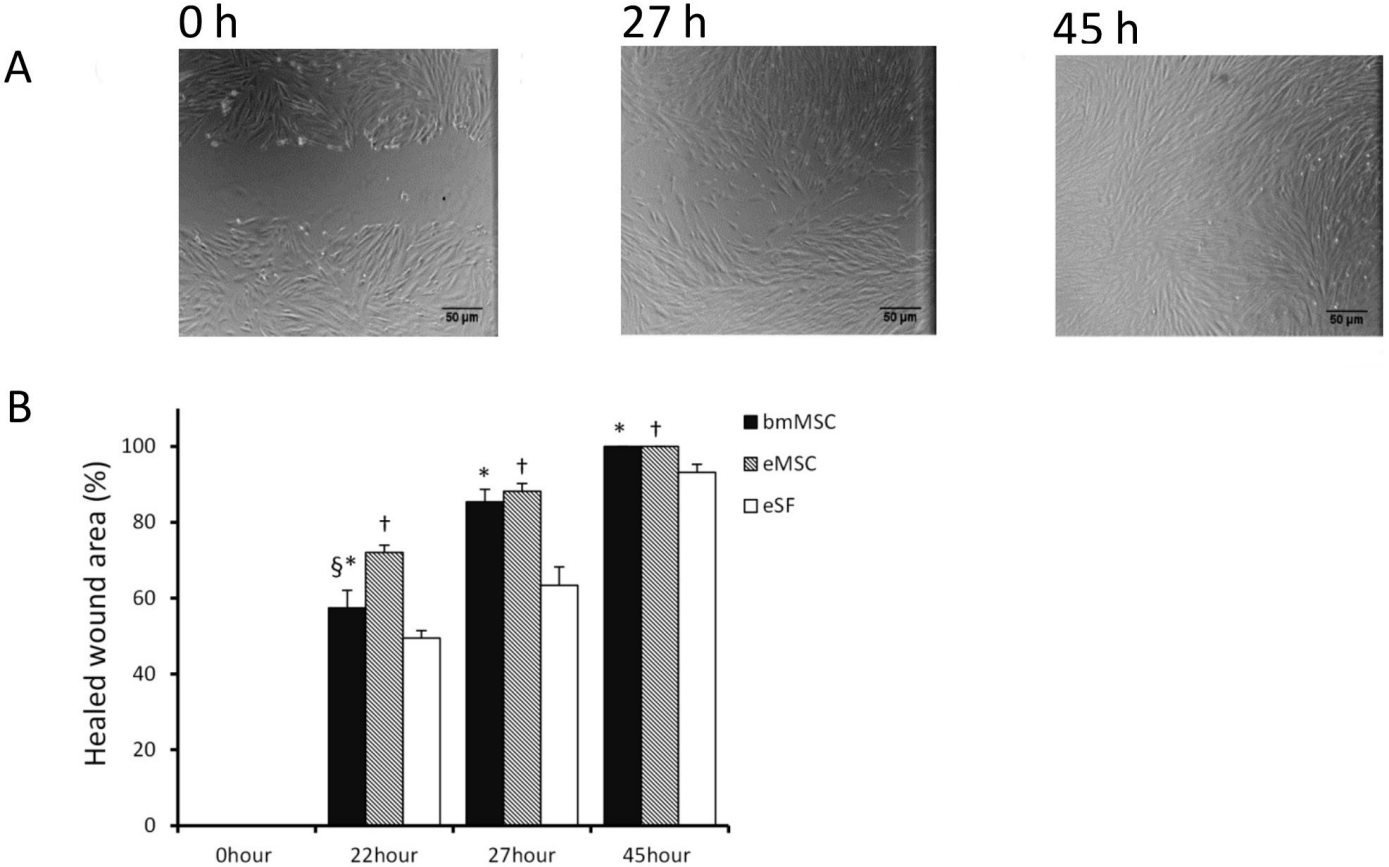
Migration/proliferation capacity of bmMSCs, eMSCs and eSFs in scratch assays at 0, 27 and 45 h. (A) Demonstration of one of the eMSCs samples at 0 h (left), 27 h (middle), and 45 h time points (right). All eMSCs reached 100% closure of the scratch by 45 h. Pictures captured at 10x magnification; scale bar 50 μm. (B) Quantitative data of closed scratch areas over time in bmMSCs, eMSCs and eSFs showing higher proliferation/migration potential in eMSCs and bmMSCs compared with eSFs. Statistical analysis: *bmMSCs vs. eSFs, ^†^eMSCs vs. eSFs, ^§^bmMSCs vs. eMSCs; p<0.05.

In the scratch assay protocol, the closing of the scratched area was measured until one of the cell types showed 100% closing of the scratch (Fig 3A). From 27 h onwards, eMSCs and bmMSCs showed similar proliferation/migration capacities (85–88%), both presenting closing of the scratch at 45 h. Interestingly, the eMSCs had the highest proliferation/migration rate compared with bmMSCs and eSFs at the early time points (22 h: 72% vs. 57% and 50%, P<0.05) (Fig 3B). The eSFs had a significantly lower proliferation/migration capacity than eMSCs and bmMSCs at all time points, but showed a confluence of up to 90% at 45 h (Fig 3B).

**Fig 4:**
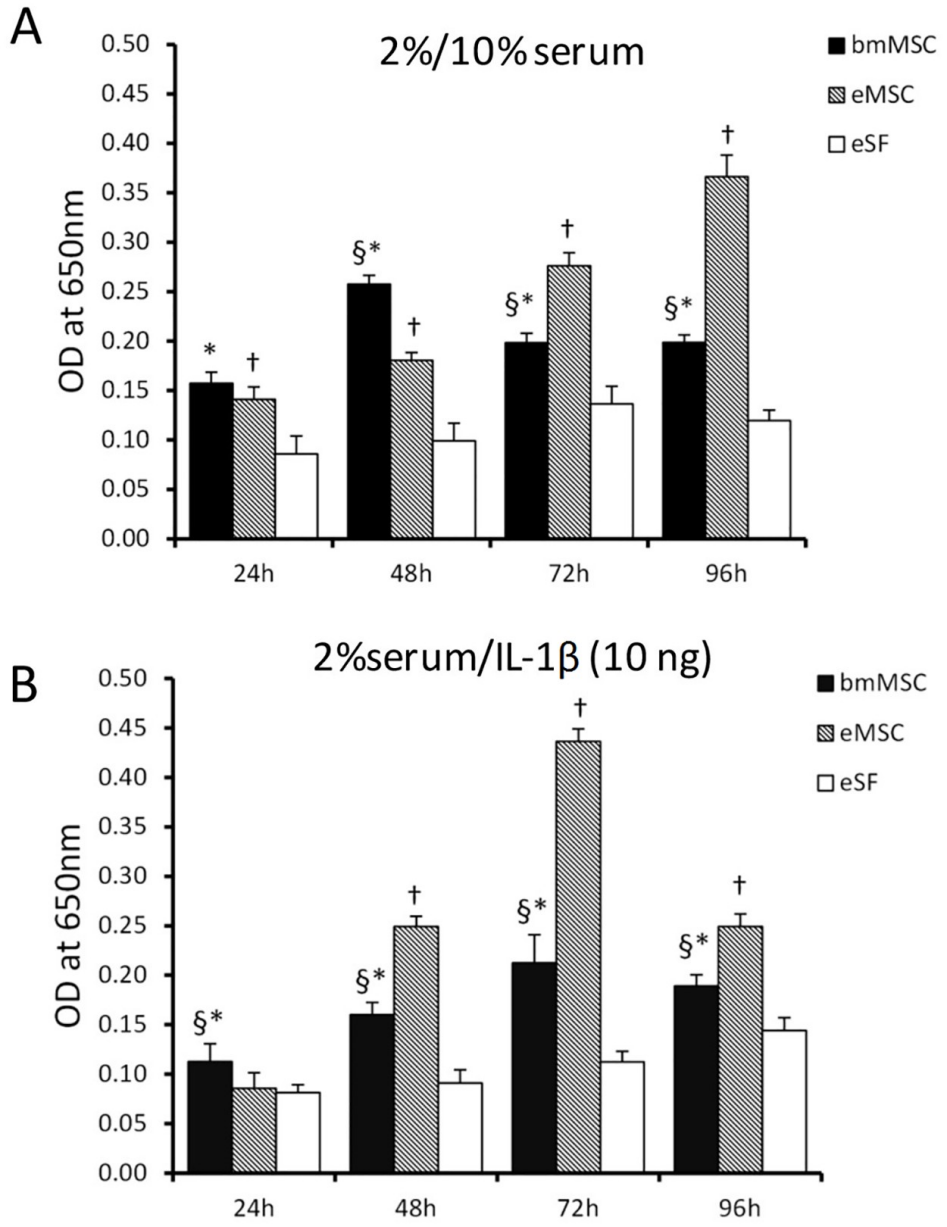
Vertical migration assays of bmMSCs, eMSCs and eSFs followed up till 96 h. (A) Using serum attractant (2% serum in upper and 10% in lower chamber), the eMSCs seemed to accelerate migration activity compared to other cell types up till 96 h (B) Inflammatory attraction was assessed by using IL-1β (10 ng/ml) in the lower chamber. The migration response was high in both stem cell population whereas no significant migration activity was observed in eSFs. ^§^ p<0.05 bmMSCs vs. eMSCs; * p<0.05 bmMSCs vs. eSFs; ^†^ p<0.05 eMSCs vs. eSFs.

After assessing the optimal vertical migration time period, the end point was set to 96 h for all cell types (Fig 4). The cell migration from 2% towards 10% serum attractant revealed the high migration potential of bmMSCs compared with endometrial cells up to 48 h (p< 0.05). However, eMSCs showed the highest migration activity at 96 h while no significant migration was observed among eSFs (Fig 4A). When the cells were subjected to cytokine chemoattraction with IL- 1β, the migration activity of bmMSCs and eMSCs increased up to 72 h. The eMSCs demonstrated the highest migration activity at 72 h with IL- 1β trigger (+125% compared with bmMSCs, +200% compared with eSFs), whereas the eSFs did not show any significant migration (Fig 4B).

### LPS-stimulated eMSCs and eSFs possess a quiescent cytokine/chemokine secretion pattern compared with bmMSCs

**Fig 5:**
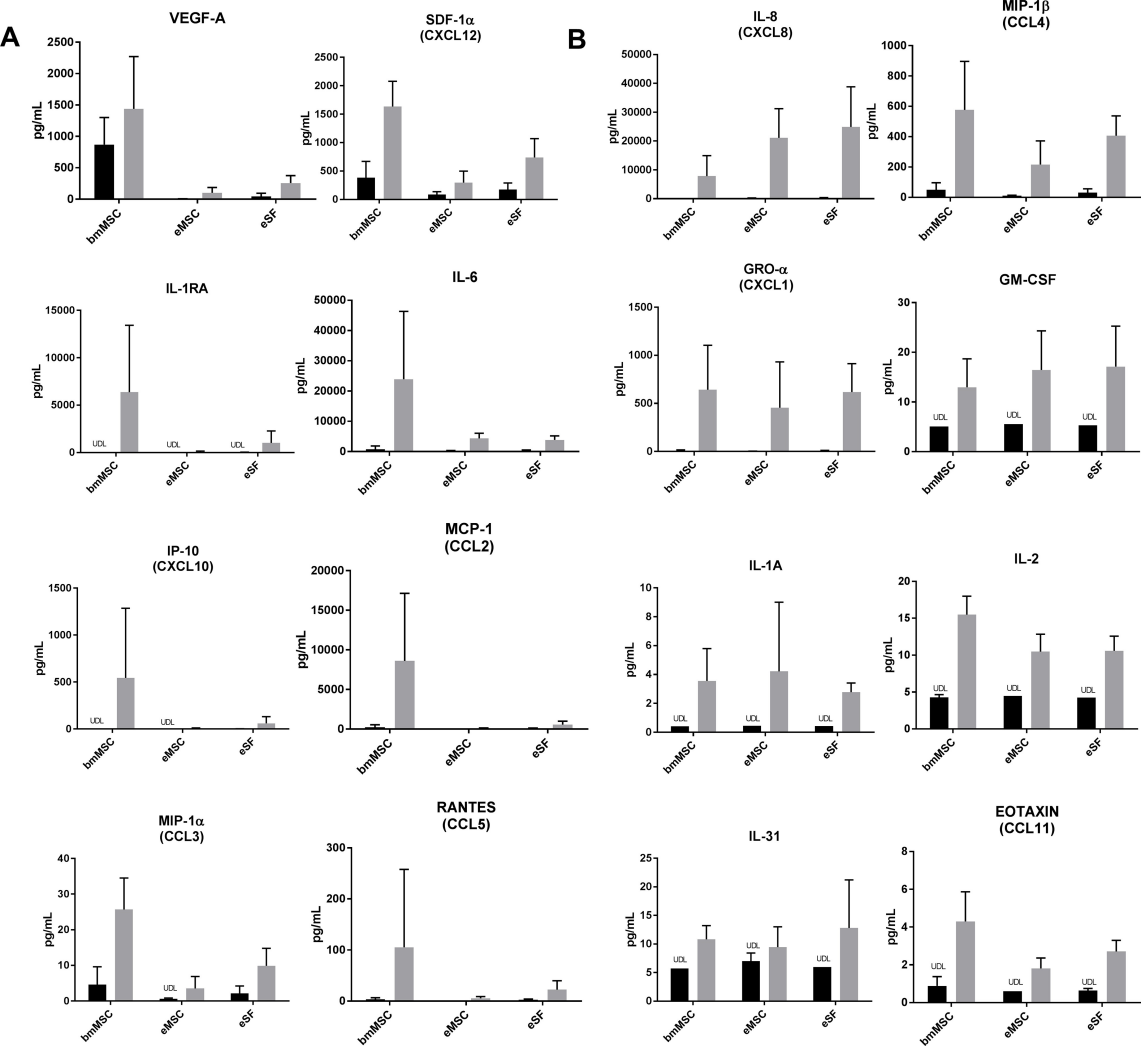
Basal and LPS-stimulated cytokine secretion analysis of bmMSCs, eMSCs and eSFs with Luminex multiplex assay. The overall cytokine pattern was shown to be pronounced in bmMSCs compared to endometrial cell types. (A) Cytokines highly expressed by bmMSCs vs. eMSCs and eSFs. (B) Cytokines having equal or higher expression in eMSCs and eSFs compared with bmMSCs. UDL = under detection limit; * p<0.05 bmMSCs vs. eMSCs or eSFs; ^#^ p<0.05 eMSCs vs. eSFs. Basal level (black), LPS stimulated (gray).

From the original 35 analytes, the following 16 analytes were detected in the Luminex analysis in a basal and/or LPS-stimulated state: VEGF-A, SDF-1α, IL-1RA, IL-6, IP-10, MCP-1, MIP-1α, RANTES, IL-8, MIP-1β, GRO-α, GM-CSF, IL-1α, IL-2, IL-31 and Eotaxin. The expression of all targets is presented in Table 2. In general, the cytokine secretion pattern of bmMSCs was pronounced compared with the endometrial cell types, and only a few differences were shown between eMSCs and eSFs. bmMSCs showed eminently higher levels of VEGF-A, SDF-1α, IL-1RA, IL-6, IP-10, MCP-1, MIP-1α and RANTES than eMSCs and/or eSFs with most of the differences being shown after LPS stimulation (p<0.05, Fig 5A, Table 2). The VEGF-A and SDF-1α secretion in bmMSCs was high at both basal levels as well as after LPS stimulation compared with eMSCs and eSFs. The basal IL-8 secretion was significantly higher in the endometrial cell populations than bmMSCs, and after LPS stimulation the secretion tended to be still lower in bmMSCs compared with endometrial cells types (p<0.05, Fig 5B, Table 2). MIP-1β, and GRO-α were similarly expressed before and after LPS stimulation in all three cell types (Fig 5B, Table 2). Interestingly, the eMSCs and eSFs showed overall a relatively low cytokine secretion pattern compared with bmMSCs, and there were only a few differences between eMSCs and eSFs showing higher expression of IL-1RA, IP-10 and RANTES in eSFs compared to eMSCs. Several cytokines undetected at the basal state, however, presented with high (IL1-RA and IP-10) or moderate response (GM-CSF, IL-1α, IL-2, IL-31 and Eotaxin) after LPS stimulation (Fig 5A and 5B, Table 2).

**Table 2:** Cytokine secretion analysis of bmMSCs, eMSCs and eSFs with Luminex multiplex assay.

Target	Detection limit (pg/ml)	Basal secretion (pg/ml ± STDEV)			LPS stimulated secretion (pg/ml ± STDEV)			p-value
		bmMSCs	eMSCs	eSFs	bmMSCs	eMSCs	eSFs	
IL-1α	0.45	UDL	UDL	UDL	3.6 ± 2.2	4.2 ± 4.8	2.8 ± 0.6	
IL-1β	0.59	ND	ND	ND	ND	ND	ND	
IL-1RA	25.46	UDL	UDL	UDL	6372 ± 7060	71.1 ± 84.6	1017 ± 1260	*, #
IL-2	4.47	UDL	UDL	UDL	15.5 ± 2.5	10.5 ± 2.4	10.6 ± 2	
IL-4	10.99	ND	ND	ND	ND	ND	ND	
IL-5	8.8	ND	ND	ND	ND	ND	ND	
IL-6	9.2	771 ± 1090	215 ± 140	310 ± 200	23939 ± 22420	4342 ± 1730	3862 ± 1320	*, ^
IL-7	0.26	ND	ND	ND	ND	ND	ND	
IL-8 (CXCL8)	2.01	7.4 ± 5.8	168 ± 110	220 ± 180	7837± 7120	21090 ± 10160	24900 ± 13900	$, "
IL-9	7.46	ND	ND	ND	ND	ND	ND	
IL-10	1.57	ND	ND	ND	ND	ND	ND	
IL-12p70	3.23	ND	ND	ND	ND	ND	ND	
IL-13	1.0	ND	ND	ND	ND	ND	ND	
IL-15	0.8	ND	ND	ND	ND	ND	ND	
IL-17A	1.87	ND	ND	ND	ND	ND	ND	
IL-18	12.6	ND	ND	ND	ND	ND	ND	
IL-21	6.93	ND	ND	ND	ND	ND	ND	
IL-22	6.94	ND	ND	ND	ND	ND	ND	
IL-23	14.81	ND	ND	ND	ND	ND	ND	
IL-27	18.67	ND	ND	ND	ND	ND	ND	
IL-31	6.3	UDL	UDL	UDL	10.8 ± 2.4	9.5 ± 3.5	12.8 ± 8.4	
TNF-α	7.02	ND	ND	ND	ND	ND	ND	
TNF-β	5.6	ND	ND	ND	ND	ND	ND	
IFN-α	0.51	ND	ND	ND	ND	ND	ND	
IFN-γ	10.82	ND	ND	ND	ND	ND	ND	
GM-CSF	5.57	UDL	UDL	UDL	13.0 ± 5.7	16.4 ± 7.9	17.1 ± 8.2	
MCP-1 (CCL2)	1.02	231 ± 310	5.0 ± 4.6	64.5 ± 67.3	8606 ± 8540	66.5 ± 67.7	551 ± 440	*, ^, $
MIP-1α (CCL3)	0.54	4.6 ± 5	UDL	2.2 ± 2	25.7 ± 8.8	3.5 ± 3.3	9.9 ± 4.9	*, ^
MIP-1β (CCL4)	1.56	49.4 ± 47	10.3 ± 3.5	31.2 ± 24.8	576 ± 320	217 ± 160	406 ± 130	
RANTES	0.54	3.3 ± 3.3	0.6 ± 0.03	2.7 ± 1.5	106 ± 150	5.6 ± 3.2	22.3 ± 17.5	*, #
EOTAXIN (CCL11)	0.6	UDL	UDL	UDL	4.3 ± 1.6	1.8 ± 0.6	2.7 ± 0.6	*
GRO-α (CXCL1)	2.57	8.5 ± 7.9	3.3 ± 0.9	6.7 ± 5.1	642 ± 460	456 ± 480	619 ± 290	
IP-10 (CXCL10)	0.9	UDL	UDL	2.0 ± 1.3	544 ± 740	6.0 ± 5.8	60.4 ± 71	*, #
SDF-1α (CXCL12)	10.15	383 ± 290	87.1 ± 48.6	175 ± 120	1634 ± 450	298 ± 200	738 ± 330	*, ^
VEGF-A	3.3	870 ± 430	5.0 ± 2.7	43.6 ± 50.9	1438 ± 830	101 ± 85.5	257 ± 120	*, ^, $, "

ND = not detected, UDL = under detection limit

* p<0.05 bmMSCs vs.eMCs after LPS stimulation

^ p<0.05 bmMSCs vs.eSFs after LPS stimulation

^#^ p<0.05 eMSCs vs.eSFs after LPS stimulation

^$^ p<0.05 bmMSCs vs.eMCs in basal state

”p<0.05 bmMSCs vs.eSFs in basal state

## Discussion

**Fig 6:**
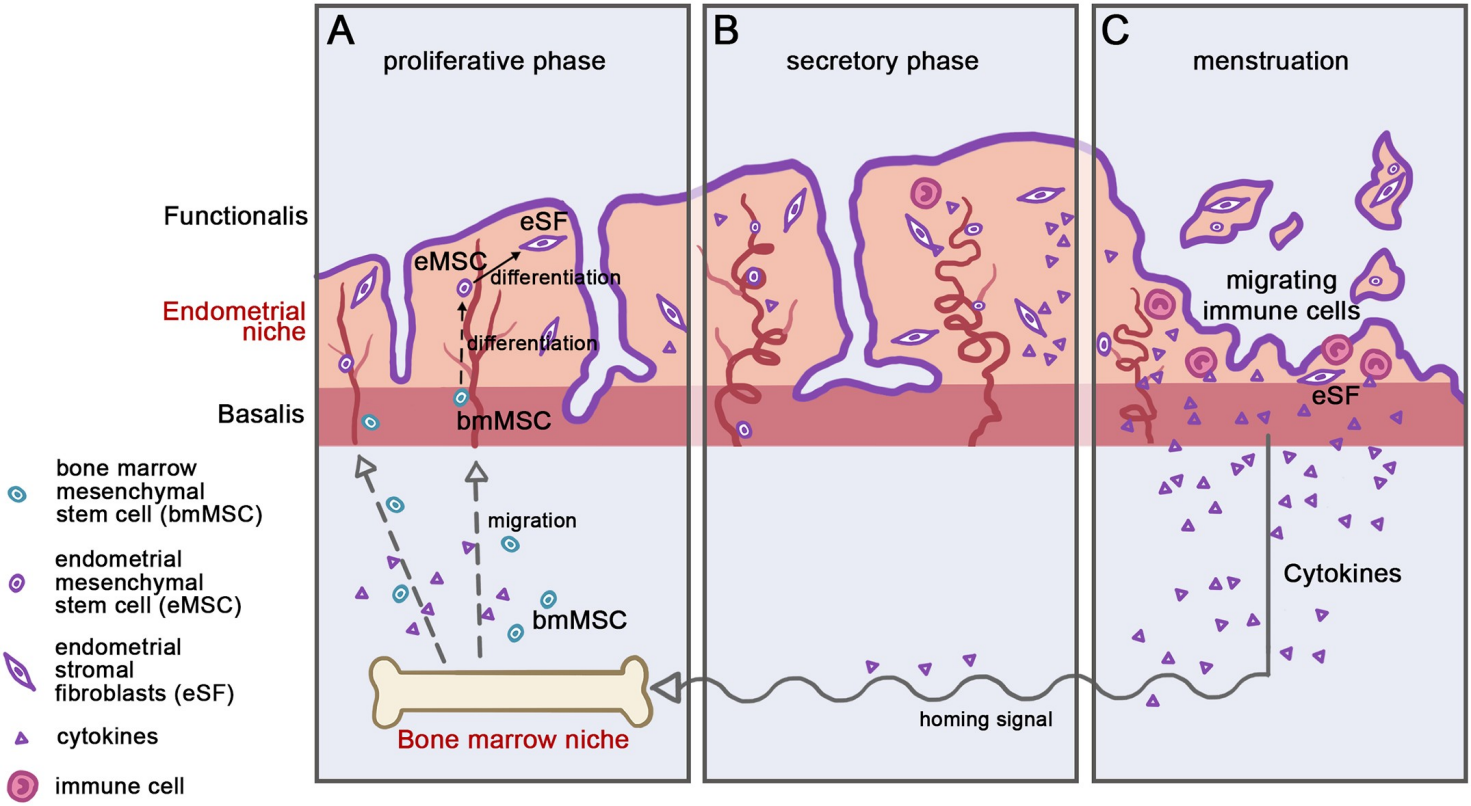
Schematic depiction of bmMSC migration to human endometrium in response to hormonal/cytokine driven homing signals. (A) The migration of bmMSCs to the human endometrium contributes to the endometrial stem cell pool (the endometrial mesenchymal stem cells, eMSCs) and thereby endometrial renewal. The change in niche and the differentiation process towards eSFs will alter the migration properties and the cytokine secretion profile of these cells. (B-C) The estrogen (E2) and progesterone (P4) effect and their withdrawal will drive endometrial collapse and subsequent hypoxia during the late secretory phase of the menstrual cycle most likely triggering the homing signal for the bmMSCs for the subsequent cycle.

This is the first study to compare the surface marker characteristics, migration potential and cytokine profiles between bmMSCs, eMSCs and eSFs in the same study setting. The results reveal that both bone marrow and endometrial stem cells share similar surface markers along with the high proliferation activity and migration potential compared to eSFs implying that differentiation process towards eSF-phenotype alters these characteristics. Interestingly, the bmMSCs show distinct differences in their cytokine secretion profiles whereas the endometrial cells have more similar profile plausibly due to sharing the similar niche compared with bmMSCs (Fig 6).

According to the previous studies, the bmMSCs serve as progenitors for endometrial eMSCs [12,39]. On the other hand, recently, the link between eMSCs and eSFs was strengthen, as these two cell types revealed similar gene expression profiles and *in vitro*, the eMSCs were shown to differentiate into eSFs [2,9]. In keeping with this, the present data showed the bmMSCs and eMSCs presenting with similar, well-known stem cell surface marker characteristics and eMSCs with high clonogenic potential compared with eSFs [1,40,41]. Furthermore, supporting the idea that the eMSCs are more likely to be of bmMSCs origin rather than direct progeny of the haematopoietic stem cell lineage, all three cell types were negative for haematopoietic cell surface antigens [42].

In accordance with the data showing stem cells being involved in different repair processes, the bmMSCs and eMSCs were found to have higher proliferation potential than differentiated eSFs. Furthermore, the stem cells also showed high migration activity, an important ability enabling homing into the site of injury in regenerative processes. Even though the endometrial stem cells are not known to have any specific role as migratory cells, the data is in line with previous study validating also high migration potential in adipose tissue driven MSCs compared to bmMSCs [43]. Furthermore, the data underline the notion that also harboured stems cells retained the migration potential that may be activated with certain triggers as also suggested to be the case for human endometrial stem cells [44]. On the other hand, the eSFs did not present with significant migration potential indicating that differentiation process towards eSF-phenotype alters the migration capacity and thus limit the number of migratory cells in human endometrium. In fact, similar to the findings by Barragan et al., in the present study the eMSCs also started rapidly differentiating *in vitro* into eSFs shown as loss of CD146 expression [9]. At Passage 2, only 70% of the eMSCs expressed CD146, indicating an active differentiation process towards eSFs even though the cells were cultured only for a relatively short time and they were not allowed to reach 100% confluence. Whether this feature relates to decreased migration activity in eMSCs cannot be concluded from the present data, however, one may hypothesize that in case of impaired differentiation process towards eSFs, as shown by Barragan et al., the retained migration ability of eMSCs might be of relevance regarding pathologies like endometriosis and adenomyosis.

The collapse of the endometrium during menstruation leads to hypoxia and a vast inflammatory cascade that most likely serve as recruitment signals for stem cells to harbour endometrium [28,45–47]. IL-1 is a potent cytokine with several functions, including a role in immune balance during the implantation process along with the regulatory role in menstruation by modulating inflammatory cascades. *Ex utero* studies have also shown that the bmMSCs are being recruited by specific growth factors/cytokines, one of them being IL-1β [48]. Given that IL-1β is abundant during menstruation and its expression is strongly linked to hypoxia, it may play as one of the endometrium-related recruiting signals for bmMSCs [26–28]. In the present data IL-1β was able to trigger high migration response both in bmMSCs and eMSCs, with eMSCs presenting even higher migration response to IL-1β than the bmMSCs. Whether this is of clinical importance in maintaining eMSCs in the endometrium is not clear but potentially high peripheral IL-1β could recruit eMSCs to ectopic locations. Moreover, recently endometrial stromal cell SDF-1 expression was shown to be involved in bmMSC recruitment where estrogen promoted the expression of SDF-1 receptor CXCR4 [46]. Given that IL-1β has been shown to promote SDF-1 expression [49], it may be that these signals together with hormonal regulation play a role driving bmMSC recruitment and harbouring to the human endometrium.

The change in niche upon stem cell migration and its effect on intrinsic inflammatory characteristics may also give insight into the differences between bmMSCS, eMSCs and eSFs. To date, only few studies have been done regarding the comparison between bmMSCs’ cytokine secretion profile to eMSCs’ or eMSC cytokine profile compared to other endometrial cell types [37,50,51] and no data exist on PDGFRβ+/CD146+ isolated eMSCs. Furthermore, most of the studies have only investigated the basal cytokine secretion profile, although it is well established that it is not sufficient to characterize the full secretion potential of these cells [30,32,33]. Indeed, in the present data, the major differences in cytokine secretion profiles between the different cell types were shown under LPS-induced inflammation. The LPS response in the cells might occur through toll-like receptor 4 (TLR4) activation shown recently in mesenchymal stem cells [52] but also in rodent and human endometrium [53,54]. The bmMSCs exhibited a robust cytokine expression of several targets (VEGF-A, SDF-1α, IL-1RA, IL-6, IP-10, MCP-1, MIP-1α and RANTES) in response to LPS stimulation compared with endometrial cell types. IL-1β, on the other hand, was not detected in either eMSC or eSF secretome, thus implying immune cells most likely being the major source for this particular cytokine during endometrial shedding [37]. The secretion of VEGF-A, a growth factor necessary for endometrium vasculature repair related to hypoxia [55–59], was demonstrated in all three cell types; however, the bmMSCs presented with higher expression than endometrial cells. This is in line with the important role of bmMSCs in regeneration processes in general [48]. Interestingly, the VEGF-A secretion pattern was not in line with the previous data by Gaafar *et al*. showing higher VEGF-A expression in endometrial cells than bmMSCs [60]. The reason for this discrepancy might be due to the fact that the eMSCs in the Gafaar´s work were obtained through culturing and not by FACS isolation, thus possibly including also other cells than mesenchymal stem cells. On the other hand, in line with previous studies, the bmMSCs were shown to produce high levels of SDF-1α [46], whereas the secretion was moderate from the endometrial cells. The SDF-1/CXCR4 signalling is considered crucial for bmMSC recruitment in general, and, as mentioned earlier, recently the endometrial stromal cell SDF-1α was also shown to attract mouse bmMSC through CXCR4 activity promoted by estrogen [46,61]. As for both VEGF-A and SDF-1 secretion profile it might be that in order to avoid abundant endometrial bmMSC migration and balanced bmMSC recruitment, the endometrium presents with more quiescent secretion profile for these pivotal signals.

IL-8 has been found to have an important role regulating the recruitment of leucocytes to the endometrium and promoting endometrial stromal viability and proliferation [62,63]. The LPS-stimulated IL-8 levels were comparable in all three cell types; however, interestingly, the basal IL-8 levels were slightly elevated in endometrial cells compared with the bmMSCs. Furthermore, some of the cytokine targets that have been demonstrated to play a role in implantation processes and correlate with the implantation success in IVF treatments were expressed evenly by bmMSCs and both endometrial cell types (MIP-1β, GRO-α) or even more robustly by bmMSCs (MCP-1, IP-10) [64,65]. These data are of importance, especially considering a recent study where bmMSCs therapy was carried out successfully for patients with thin endometrium leading to the endometrial repair and live birth [13]. As expected, a subset of cytokines was not detected at the basal level, but their secretion was triggered by the inflammatory stimuli extending the cytokine profile of these cells (IL-1RA, IP-10, GM-CSF, IL-1α, IL-2, IL-31 and Eotaxin). From these specifically ILR1A, IP-10, GM-CSF and EOTAXIN are all shown to be involved in implantation process controlling trophoblast cell attraction/invasion [66–70]. Altogether, one can speculate that the subtle cytokine expression pattern of the endometrial cell types compared with the bmMSCs might serve normal endometrial function by facilitating a balanced endometrial niche and by providing a non-hostile environment for the implanting embryo.

The strength of the study lies in the rigorously collected rare human sample depository with paired endometrial samples. Although obtaining bmMSCs and endometrial cell population from the same patient would result into even more rigorous data, in practice this was not feasible. As MSCs have been shown to lose some of their properties with ageing [71,72], only samples from reproductive-aged women were included. Regarding the endometrial cell types, Schwab et al. demonstrated that FACS sorting of the eMSCs with PDGFRβ+/ CD146+ double staining yielded cells with a more stem cell-like phenotype compared to magnetic sorted eMSCs [1]. Thus, in our study, the purity of endometrial cell types was enhanced by FACS isolation with PDGFRβ/CD146-labeling. There are also several limitations that need to be addressed. Due to the small sample size, the study is considered as a pilot *in vitro* study that lays ground for future experiments regarding the investigations on endometrial renewal and endometrium-related pathologies like endometriosis and adenomyosis. Moreover, the hormonal and paracrine secretion or the interaction of other endometrial cells types on the metabolic/immunuosecretory properties of MSC populations was not assessed. As for the migration assay, the effects of other cytokines/chemokines and growth factors or the synergistic effects of other endometrial cell types cannot be concluded.

## Conclusions

This is the first study including bmMSCs, eMSCs and their progeny eSFs in the same study setting, allowing the simultaneous and more precise comparison of these three cell types regarding their proliferative, migratory and inflammatory characteristics. The data showed similar surface marker profiles and high migration potential towards inflammatory attractants in bmMSCs and eMSCs, while the endometrial cell population sharing similar niche had distinct, subtler, cytokine profile compared with bmMSCs. While bmMSCs’ high proliferation and migration potential supports their role in different renewal processes, including human endometrium, one can hypothesize, that the change in the cytokine secretion profile along differentiation process towards eSFs might enable a subtler cytokine profile possibly contributing to normal endometrial function.
